# Association between duration of coronary occlusion and high-intensity signal on T1-weighted magnetic resonance imaging among patients with angiographic total occlusion

**DOI:** 10.1007/s00330-016-4672-0

**Published:** 2017-02-02

**Authors:** Kenji Matsumoto, Shoichi Ehara, Takao Hasegawa, Mikumo Sakaguchi, Kenei Shimada

**Affiliations:** Department of Cardiovascular Medicine, Osaka City University Graduate School of Medicine, 1-4-3 Asahi-machi, Abeno-ku, Osaka 545-8585 Japan

**Keywords:** Chronic total occlusion, Coronary artery disease, Magnetic resonance imaging, Atherosclerotic plaque, Thrombosis

## Abstract

**Objectives:**

To evaluate the association between duration of the coronary occlusion and high-intensity signal (HIS) on noncontrast T1-weighted imaging using a 1.5-T magnetic resonance imager among patients with angiographic coronary total occlusion.

**Methods:**

The signal intensity of the coronary target area divided by the signal intensity of the left ventricular muscle near the target area at each site (TMR) was measured. Areas with a TMR >1.0 were defined as HIS. Thirty five lesions from 33 patients were divided into the following three groups: subacute occlusion (up to 3 months; *n* = 7), short-duration chronic total occlusion (SD-CTO: 3–6 months; *n* = 9) and long-duration CTO (LD-CTO: ≥6 months; *n* = 19).

**Results:**

All subacute occlusion lesions showed a HIS within the occlusion site. Among patients with CTO, the frequency of a HIS within the occlusion site was significantly higher in SD-CTO than in LD-CTO lesions (*p* = 0.013). In multivariate analyses, only an occlusion duration of less than 6 months was an independent factor associated with the presence of HIS (odds ratio 7.6, 95% CI 1.1–54.5; *p* = 0.044).

**Conclusions:**

The presence of a HIS in the occlusion site was associated more with SD-CTO than with LD-CTO among patients with CTO.

***Key Points*:**

• *All subacute occlusion lesions show a high-intensity signal on T1-weighted imaging.*

• *HIS within occlusion sites is associated with subacute or short-duration total occlusion.*

*• T1-weighted imaging for coronary total occlusion may be useful for intervention strategy.*

## Introduction

Patients with a chronic total occlusion (CTO) are often managed conservatively or surgically compared to those with percutaneous coronary intervention (PCI). Several studies have shown that compared with conservative management, successful PCI for CTO provides significant benefits, represented by improvement of left ventricular contractile function and myocardial perfusion, and thus is associated with improved long-term clinical outcomes [1, 2]. However, coronary CTO is still the most challenging lesion for PCI, with a procedural success rate ranging from 60 to 70% compared with 98% for non-CTO PCI [1].

In cases of acute or subacute myocardial infarction, the occluding thrombus is disorganized, relatively soft, and easily crossed with a guide wire [3]. However, the process and time course of CTO thrombus morphology development remain unknown. Results from a recent pathology study showed that the short-duration CTO (SD-CTO) was characterized by abundant organized thrombi and necrotic cores, while the lumen in long-duration CTO (LD-CTO) predominantly consisted of collagen and calcification [4]. Olivari et al. reported that, with regards to CTO duration, PCI failures increased only in patients with CTOs lasting more than 6 months [5].

Cardiac magnetic resonance (MR) can noninvasively determine infarct size, transmurality and area at risk, with late gadolinium enhancement and T2-weighted images, after acute myocardial infarction (AMI) [6]. Moreover, the introduction of noncontrast T1-weighted imaging (T1WI) in MR has facilitated plaque imaging based on the presence of a high-intensity signal (HIS) within the thrombus or intraplaque haemorrhage caused by methaemoglobin T1 shortening [7–9]. Recently, some studies have demonstrated that MR imaging is able to identify not only the presence but also the stage of thrombus or haemorrhage in patients with deep vein thrombosis [10], pulmonary embolism and complex carotid plaques [7, 11]. The precise assessment of recent plaque thrombosis or haemorrhage in the coronary occlusion site may influence procedural success rates for CTO, which could lead to more appropriate risk stratification and treatment. We therefore investigated the association between the duration of coronary occlusion and presence of a HIS on T1WI on cardiac MR among patients with total angiographic occlusion.

## Materials and methods

### Patients

A total of 44 consecutive patients with total angiographic occlusion, defined as interruption of vessel continuity with a Thrombolysis in Myocardial Infarction (TIMI) flow grade score of 0, were prospectively enrolled in this study between March 2011 and December 2014. Patients with a history of prior PCI, AMI within 72 h of symptoms onset or with contraindications for MR were excluded from the study. CTO lesions in which a bypass graft was anastomosed distal to the CTO were also excluded. Coronary angiography (CAG) and cardiac MR were performed within 6 days (2 ± 1 days). Of the 44 patients initially enrolled, six were excluded from the analysis because of insufficient MR image quality (5/32 (16%) with a 5-element and 1/12 (8%) with a 32-element cardiac coil; *p* = 0.99).

Thus, 42 lesions from 38 patients were examined in this study. Here, we suggest three levels of certainty for the occlusion duration, according to the previous reports [2, 12]: (a) ‘certain’ (angiographically confirmed), in lesions where a previous angiogram confirmed the presence of TIMI 0 flow prior to the procedure; (b) ‘likely’ (clinically confirmed), objective evidence of an AMI in the territory of the occluded artery without other possible culprit arteries before the current angiogram; (c) ‘undetermined’, TIMI 0 flow and angiographic anatomy suggestive of long-standing occlusion with stable anginal symptoms unchanged or evidence of silent ischemia. Seven lesions from five patients with ‘undetermined’ occlusion duration were excluded in this study. Finally, on the basis of the ‘certain’ and ‘likely’ occlusion duration, 35 lesions from 33 patients with total occluded artery were further divided into subacute occlusion (up to 3 months since a clinical episode indicating acute occlusion; *n* = 7), short-duration CTO (SD-CTO: 3–6 months; *n* = 9) and long-duration CTO (LD-CTO: ≥6 months; *n* = 19), according to the estimated duration of total occlusion.

The study was approved by the hospital’s ethics committee, and informed consent was obtained from all patients before the study.

### Angiographic analysis of potential variables associated with successful guide wire crossing

Coronary angiograms were analysed by a single observer who was blinded to the clinical information. The angiographic morphology of the entry point was classified as tapered if the occluded segment ended in a funnel-shaped form or blunt if it did not. The presence of calcification, bending (at least one bend of greater than 45°) in the entry or body of CTO, bridging collaterals, and side branches within 3 mm proximal to the entry stump were evaluated [13]. The length of the occlusion segment was categorized as either less than 20 mm or at least 20 mm according to the consensus of the EuroCTO Club [12].

### MR coronary image acquisition

Coronary plaque imaging was performed by using a 1.5-T MR imager (Achieva, Philips Medical Systems, Best, the Netherlands) with a 5- or 32-element cardiac coil. Nitroglycerin (0.3 mg) was administered sublingually to patients immediately before taking images to obtain high-quality MR images. There were no patients with contraindication to nitroglycerin. Breath-hold transaxial cine MR images were then acquired using a steady-state free precession sequence to determine the trigger delay time when the motion of the right coronary artery was minimal. First, to obtain detailed information on the location of the target lesion, free-breathing, steady-state, free-precession, whole-heart coronary MR angiographic images were obtained [9]. Next, coronary plaque images were obtained while the patients were breathing freely, by using a three-dimensional T1WI, inversion-recovery, gradient-echo technique with fat-suppressed and radial *k*-space sampling in the *Y*–*Z* plane (repetition time, 4.4 ms; echo time, 2.0 ms; flip angle, 20°; SENSE factor, 2.5; number of excitations, 2; navigator gating window of ±1.5 mm with diaphragm drift correction; field of view, 300 × 240 × 120 mm [rectangular field of view, 80%]; acquisition matrix, 224 × 224; reconstruction matrix, 512 × 512 × 140, resulting in an acquired spatial resolution of 1.34 × 1.34 × 1.7 mm reconstructed to 0.6 × 0.6 × 0.85 mm) [9]. The patient-specific inversion time of the inversion-recovery sequence was adjusted to null blood signal by using a Look-Locker sequence.

### MR coronary signal intensity analysis

**Fig. 1:**
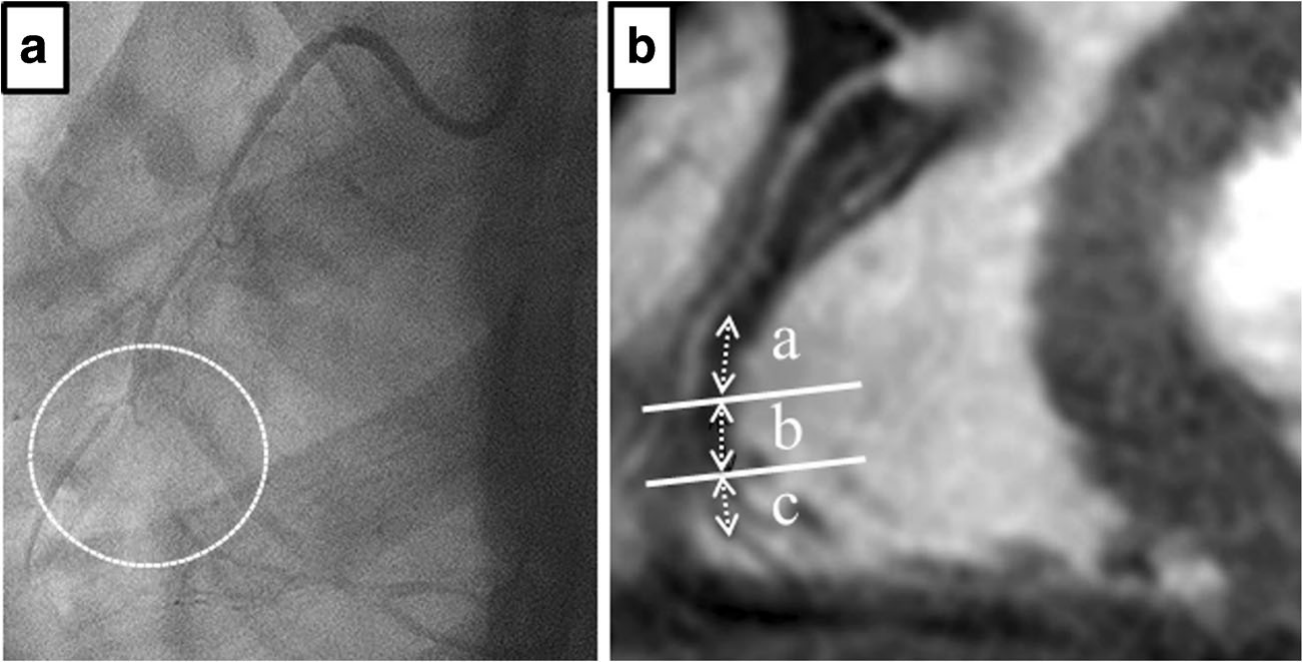
Definition of each part of the total occluded coronary arteries. **a** Bilateral CAG showing total coronary occlusion (*circle*) at the middle of the right coronary artery (RCA). **b** Whole-heart coronary MR angiography. The area corresponding to the total occlusion lesion shows a lack of blood signal (*b*) at the middle of the RCA. The target lesion was divided into three sections: *a* proximal (5 mm proximal to the occlusion site), *b* occlusion site (without signals in MR angiography), *c* distal site (5 mm distal to the occlusion site), and carefully compared with CAG

The location of the target lesion was determined by carefully comparing the CAG and MR angiographic images, by using fiduciary points such as side branches. Once the target lesion was confirmed on coronary MR angiography, the areas corresponding to the above site in the coronary T1WI obtained were carefully matched according to the surrounding cardiac and chest wall structures. Then, the target lesion in patients with CTO was visually divided into three sites by carefully comparing the bilateral CAG and MR angiographic images: (1) proximal site (within 5 mm proximal to the proximal occlusion edge), (2) occlusion site (lack of blood signal in MR angiography) and (3) distal site (within 5 mm distal to the distal occlusion edge) (Fig. 1). For further information on the lesion site, both MR angiographic images and T1WI were fused and rendered on a commercially available workstation (Ziostation 2, Ziosoft, Tokyo, Japan). Because the registration on a workstation may cause errors in determining the location of HIS (proximal site, occlusion site or distal site) due to different spatial resolution, the fused images were used just as a guide. In patients with subacute occlusion, only the occlusion site was evaluated, because the distal edge of the occlusion was difficult to identify in such lesions.

The signal intensity of the target area within a coronary artery divided by the signal intensity of the left ventricular muscle near the coronary target area at each site (TMR) was measured by placing a circular region of interest on a standard console of the clinical MR system. Areas with a TMR greater than 1.0 were defined as HIS, whereas areas with a TMR of 1.0 or lower were defined as non-HIS, according to the method described previously [8, 9]. The MR coronary image dataset was analysed by two experienced cardiologists who were blinded to the clinical information. In case of disagreement, consensus was reached by an additional joint reading. The interobserver and intraobserver coefficients of variation in the measurement of TMR were reported in our previous study [9], while the κ values for inter- and intraobserver agreements with regard to the presence of HIS in each site were 0.88 and 0.82, respectively.

### Statistical analyses

Continuous data were presented as mean ± SD. Categorical variables were compared using the χ^2^ test or Fisher’s exact test. To compare TMR differences between each group of patients with a HIS in each occlusion site, a one-way analysis of variance with subsequent Tukey-Kramer post hoc analysis was used. Interobserver and intraobserver agreement for the interpretation of HIS as the culprit lesion was performed using a κ test. Independent predictors for a HIS within the occlusion site were identified by entering age, gender and all variables associated with a *p* value less than 0.10 at univariate analysis into a logistic regression analysis. The odds ratios and 95% confidence intervals for significant independent variables in multivariate analysis were calculated. All statistical analyses were performed using SPSS version 22.0 software (SPSS, Chicago, IL, USA), with *p* values less than 0.05 considered significant.

## Results

Of the 33 patients, 5- or 32-element cardiac coil was used in 26 (79%) and 7 (21%) patients, respectively. The mean acquisition times for MR angiography and plaque imaging were 10 ± 3 min and 16 ± 3 min, respectively. Clinical characteristics and angiographic findings of all patients are shown in Table 1. Mean age of the study cohort was 70 ± 11 years, and most patients were men. The mean left ventricular ejection fraction was 45% and 16 (49%) patients had advanced chronic kidney disease (estimated glomerular filtration rate less than 60 mL/min/1.73 m^2^). Seven (21%), 2 (6%), 16 (49%) and 8 (24%) patients experienced recent myocardial infarction, unstable or stable angina pectoris, and silent myocardial ischemia, respectively.

**Table 1:** Patient clinical characteristics and angiographic findings

	(*n* = 33)
Age (years)	70 ± 11
Male	30 (91%)
Hypertension	24 (73%)
Dyslipidaemia	19 (58%)
Diabetes mellitus	13 (39%)
Smoking history	23 (70%)
LVEF %	45 ± 11
eGFR <60 mL/min/1.73 m^2^	16 (49%)
Prior MI	11 (33%)
Prior CABG	2 (6%)
Diagnosis	
Recent MI	7 (21%)
UAP	2 (6%)
SAP	16 (49%)
SMI	8 (24%)
Number of diseased vessels	
1	16 (49%)
2	14 (42%)
3	3 (9%)

Values are mean ± SD, *n* (%)

*LVEF* left ventricular ejection fraction, *eGFR* estimated glomerular filtration rate, *MI* myocardial infarction, *CABG* coronary artery bypass graft surgery, *UAP* unstable angina pectoris, *SAP* stable angina pectoris, *SMI* silent myocardial ischemia

Table 2 shows the baseline angiographic and procedure characteristics among lesions with CTO and subacute occlusion. Overall, the culprit vessel in CTO lesions was the left anterior descending coronary artery, left circumflex coronary artery and right coronary artery in 12 (43%), 5 (18%) and 11 (39%) patients, respectively. Fifteen (79%) of 19 LD-CTO lesions showed a blunt stump at entry. Procedural PCI success rates were 56% for SD-CTO and 37% for LD-CTO, while those for lesions with subacute occlusion were 100%.

**Table 2:** Angiographic and procedural characteristics

	CTO			Subacute occlusion (*n* = 7)
	Overall (*n* = 28)	SD-CTO (*n* = 9)	LD-CTO (*n* = 19)	
Culprit vessel				
LAD	12 (43%)	5 (56%)	7 (37%)	2 (29%)
LCx	5 (18%)	0	5 (26%)	2 (29%)
RCA	11 (39%)	4 (44%)	7 (37%)	3 (42%)
CTO lesion characteristics				
Side branches	25 (89%)	9 (100%)	16 (84%)	―
Blunt stump type at entry	19 (68%)	4 (44%)	15 (79%)	―
Calcification	12 (43%)	4 (44%)	8 (42%)	―
Bridge collateral	10 (36%)	3 (33%)	7 (37%)	―
Bending	7 (25%)	3 (33%)	4 (21%)	―
Occlusion length ≥20 mm	17 (61%)	6 (67%)	11 (58%)	―
Procedure after CAG				
PCI				
Successful	12 (43%)	5 (56%)	7 (37%)	7 (100%)
Unsuccessful	9 (32%)	3 (33%)	6 (32%)	0
CABG	2 (7%)	1 (11%)	1 (5%)	0
OMT	5 (18%)	0	5 (26%)	0

Values are *n* (%)

*CTO* chronic total occlusion, *SD* short duration, *LD* long duration, *LAD* left anterior descending coronary artery, *LCx* left circumflex coronary artery, *RCA* right coronary artery, *CAG* coronary angiography, *PCI* percutaneous coronary intervention, *CABG* coronary artery bypass graft surgery, *OMT* optical medical therapy

**Fig. 2:**
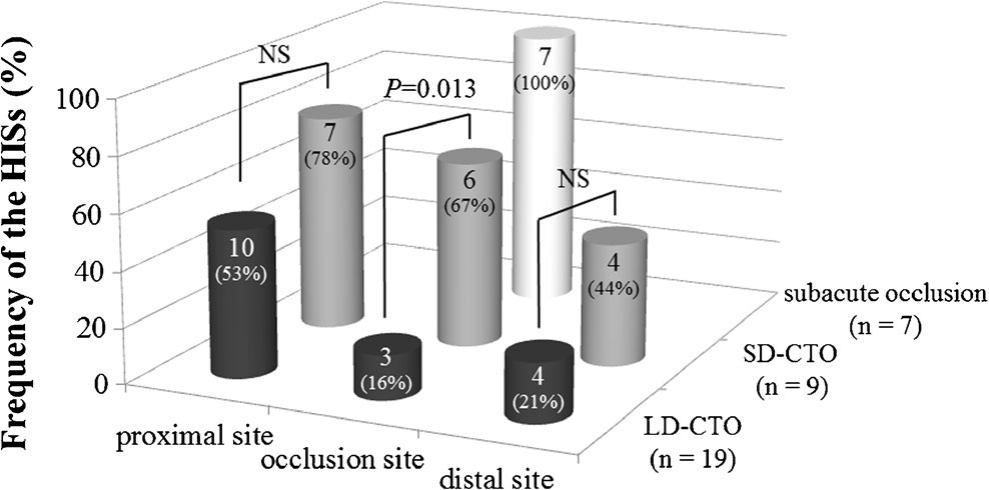
Frequency of HISs in the three occlusion lesion sections (proximal, occlusion site and distal) in a subgroup of lesions with subacute occlusion, SD-CTO and LD-CTO

Figure 2 shows the frequency of HISs at three sites (occlusion, proximal and distal) in a subgroup of subacute occlusion, SD-CTO and LD-CTO lesions. Interestingly, all subacute occlusion lesions showed a HIS within the occlusion site. However, HISs in the proximal, occlusion and distal sites of SD-CTO and LD-CTO lesions were observed in 7 (78%), 6 (67%) and 4 (44%), and in 10 (53%), 3 (16%) and 4 (21%), respectively. Among patients with CTO, the frequency of a HIS within the occlusion site was significantly higher in SD-CTO than in LD-CTO lesions (*p* = 0.013). The presence of a HIS as a predictor of SD-CTO had a sensitivity, specificity, and positive and negative predictive values of 67%, 84%, 67% and 84%, respectively. However, no significant differences in the frequency of HIS between SD-CTO and LD-CTO lesions at both proximal and distal sites were observed. A HIS was more frequently found at the proximal site of the occlusion edge.

**Fig. 3:**
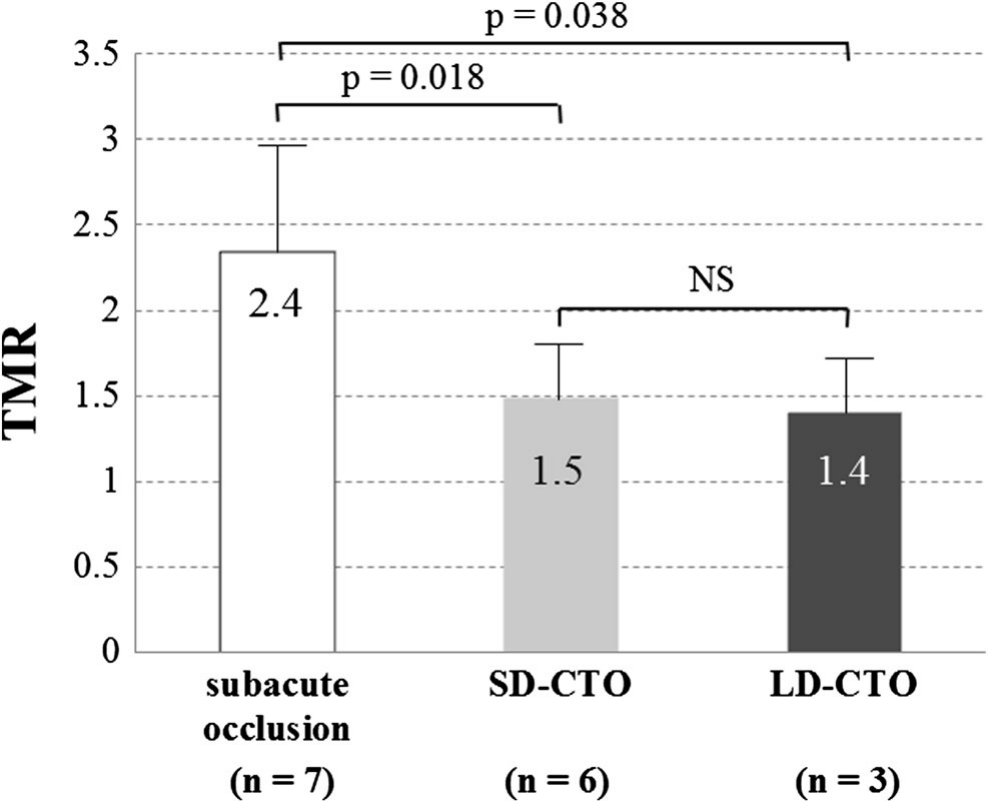
TMR according to occlusion duration among patients with a HIS in the occlusion site

Differences in TMR between groups in patients with a HIS in the occlusion site were observed (Fig. 3). The mean TMR in patients with subacute occlusion, SD-CTO and LD-CTO was 2.4 ± 0.6, 1.5 ± 0.3 and 1.4 ± 0.3, respectively. In multiple comparisons, the TMR in patients with subacute occlusion was significantly higher than that of SD-CTO (*p* = 0.018) and LD-CTO (*p* = 0.038).

Among SD- and LD-CTO lesions, multivariate logistic regression analysis to identify independent factors associated with HIS within the occlusion site (Table 3) showed that only an occlusion duration of less than 6 months was independently associated with the presence of HIS.

**Table 3:** Univariate and multivariate analysis for association with HIS in the occlusion site

Factors	Univariate analysis			Multivariate analysis		
	OR	95% CI	*P* value	OR	95% CI	*P* value
Age	1.04	0.96–1.1	0.34	1.05	0.96–1.2	0.33
Male	0.19	0.02–2.5	0.23	0.56	0.02–17.6	0.74
Side branches	0.94	0.07–12.0	0.99			
Blunt stump type at entry	0.21	0.04–1.2	0.097	0.27	0.03–2.3	0.23
Calcification	1.10	0.22–5.4	0.99			
Bridge collateral	0.86	0.16–4.6	0.99			
Bending	0.27	0.03–2.7	0.37			
Occlusion length ≥20 mm	0.73	0.15–3.7	0.99			
Duration <6 months	10.7	1.7–68.2	0.013	7.6	1.1–54.5	0.044

*HIS* high intensity signal, *OR* odds ratio, *CI* confidence interval

**Fig. 4:**
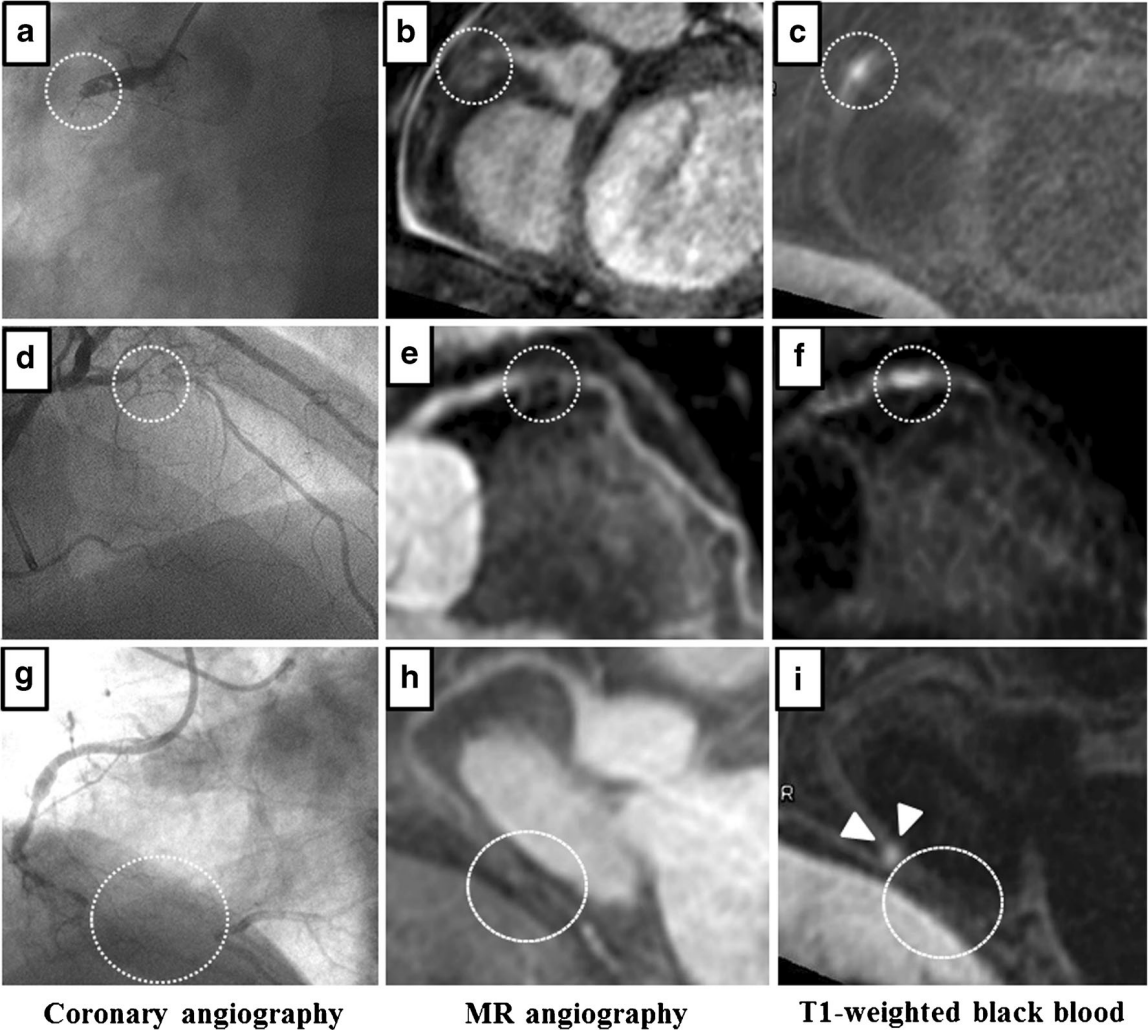
Representative angiographic images from patients with total coronary occlusion. **a**–**c** Coronary subacute total occlusion in a patient with an occlusion estimated duration of 14 days. **a** Total occlusion of the proximal right coronary artery (RCA) demonstrated on CAG (*dotted circle*). **b** Whole-heart MR coronary angiography showing absence of a blood signal at the same site (*dotted circle*) and a poor blood signal at the distal RCA from a collateral artery. **c** A HIS is shown at the occlusion site in a T1-weighted black-blood image (*dotted circle*). **d**–**f** SD-CTO in a patient with an occlusion estimated duration of 90–120 days. **d** Total occlusion of the mid left anterior descending artery (LAD) demonstrated on CAG (*dotted circle*). **e** Whole-heart MR coronary angiography revealed a lack of blood signal at the same site (*dotted circle*), whereas a good blood signal from a collateral artery was detected at the distal LAD site. **f** T1-weighted black-blood image demonstrated HIS at the region in interest (*dotted circle*). **g**–**i** LD-CTO in a patient with an occlusion estimated duration of more than 2 years. **g** CAG revealed a total occlusion at the distal RCA site (*dotted circle*). **h** The blood signal was absent in the same lesion on whole-heart coronary MR angiography (*dotted circle*), whereas the posterior descending artery was visible because of the presence of a collateral artery. **i** Absence and presence of a HIS at the occlusion (*dotted circle*) and proximal sites (*arrowheads*), respectively

Representative images from a patient with a subacute total coronary occlusion are shown in (Fig. 4a–c). Based on clinical symptoms, the estimated duration of the occlusion was 14 days in this patient. CAG demonstrated total occlusion of the proximal right coronary artery. Whole-heart coronary MR angiography also showed the absence of a blood signal at the same site, and a poor blood signal of the distal right coronary artery from a collateral artery. The area corresponding to the occluded site shows a HIS on T1WI. Furthermore, in a patient with SD-CTO (Fig. 4d–f), both whole-heart coronary MR angiography and CAG showed a total occlusion lesion of the mid left anterior descending coronary artery. A HIS on T1WI was found at the occlusion site. However, in a patient with a LD-CTO lesion (Fig. 4g–i), total occlusion of the distal right coronary artery was detected on CAG and MR angiography. Although a HIS was present at the proximal site of the occlusion edge, no HIS was found in the occlusion site.

## Discussion

To the best of our knowledge, this is the first study to investigate an association between the duration of coronary occlusion and HIS on T1WI by cardiac MR among patients showing total coronary occlusion on angiography. The frequencies of HIS within the occlusion site of subacute occlusion, SD-CTO and LD-CTO lesions were 100%, 67% and 16%, respectively. Moreover, in patients with a HIS in the occlusion site, the TMR among those with subacute occlusion was significantly higher than that of SD-CTO and LD-CTO. On the other hand, in patients with CTO in whom the occlusion duration could be estimated, multivariate analysis revealed that the presence of HIS within the occlusion site was significantly associated with SD-CTO.

Successful PCI for CTOs has the potential to improve cardiac function and long-term survival, especially in patients in whom complete revascularization is achieved [1, 2]. Multiple modality imaging may have an impact on PCI strategy. Recently, coronary computed tomography angiography demonstrated that short CTO duration was an independent predictor for PCI success in addition to such anatomical findings as presence of marked calcification, long occlusion length and excessive vessel tortuosity [14]. However, the use of contrast material is a critical issue for patients with allergy, bronchial asthma and severe chronic kidney disease. Therefore, this noninvasive MR imaging using T1WI for coronary total occluded lesions, which has the potential to identify subacute occlusion or SD-CTO, may be useful in the assessment of PCI strategy in the interventional clinical field.

Recently, Tan et al. revealed that MR imaging is a precise and reproducible method to distinguish acute ipsilateral recurrent deep vein thrombosis from an at least 6-month-old chronic residual thrombus in the leg veins when recurrence is not suspected [10]. Moreover, Chu et al. showed that multicontrast MR images can detect and classify a carotid intraplaque haemorrhage into three stages: fresh (less than 1 week), recent (1–6 weeks) and old (more than 6 weeks) [11]. Thus, MR imaging using T1WI allows the in vivo staging of intraluminal thrombus or intraplaque haemorrhages. Although this signal appears to persist for several weeks, the overall period is less than 6 months, and the information obtained might provide data about thrombus or intraplaque haemorrhage characteristics such as its age [15]. In this study, all patients with subacute occlusion had a HIS in the occlusion site, while in patients in whom the CTO occlusion duration could be estimated, the presence of HIS was significantly and more strongly associated with SD-CTO than LD-CTO. Altogether, our findings may suggest that differences in the stage of thrombus and intraplaque haemorrhage within the occluded coronary arteries potentially reflect a HIS on T1WI.

Although there was no difference in the TMR between SD-CTO and LD-CTO among the patients with HIS in the occlusion site, the TMR in patients with subacute occlusion was significantly higher than that of CTO. In this study, all patients with subacute total occlusion were diagnosed with recent myocardial infarction. Coronary thrombus developed on the top of a disrupted vulnerable plaque in total acute coronary artery occlusion lesions mainly contains a larger amount of fibrin in a meshwork, trapping a large number of erythrocytes [16]. Kelly et al. reported that erythrocytes containing methaemoglobin produced T1 shortening, the extent of which was proportional to the level of methaemoglobin [15]. This may explain why the TMR values in patients with subacute occlusion were significantly higher than those of CTO lesions among patients showing a HIS. Moreover, these results may be also useful for PCI strategy in the patients with coronary total occluded lesion, because TMR in the occlusion site has the potential to identify subacute occlusion in addition to the absence or presence of HIS. It is well known that the occurrence of no-reflow during PCI has been shown to be associated with worse short- and long-term clinical outcomes. It occurs in 10–15% of PCI performed in patients with subacute coronary occlusion, probably because the lesions are friable, rich in thrombi and release vasoactive agents [17]. Therefore, the lesions with high TMR (especially 2.0 or greater) are likely to more vulnerable morphology represented by subacute occlusion. Recent T1WI studies showing that patients with high TMR suffered from no-reflow and myocardial injury in elective PCI supported this hypothesis [18]. Regarding the PCI in high-risk patients for no-reflow, previous studies showed that distal protection devices were beneficial and reduced microcirculation damage and left ventricular dysfunction [19]. Accordingly, considering the PCI strategy for patients with high TMR, distal protection devices may be a feasible strategy to prevent the no-reflow phenomenon and myocardial damage.In the present study, HIS was more frequently found at the proximal site to the occlusion edge. A HIS on T1WI might have arisen from the presence of a fresh thrombus at the proximal site or the existence of the high activity vulnerable plaque that might cause the vessel occlusion. From another point of view, this might be derived from the derangement of blood flow at the proximal site. As the inversion time for a null blood condition using a Look-Locker sequence was determined by adjusting to the flowing blood, the longitudinal magnetization of stagnant blood is less than that of flowing blood. Therefore, there is another possibility that the HIS at the proximal site might result from a gap in a null point.

This study has several limitations. First, as an inversion-recovery gradient-echo sequence is used for the T1WI, issues with spatial resolution and partial volume effect could provide difficulties to detect the exact location of HIS. Nevertheless, the κ value for this finding between investigators was sufficiently high. Second, we did not performed analysis using time as a continuous variable, because to determinate the precise occlusion duration is difficult when serial angiograms are unavailable and it relies largely on clinical history and electrocardiogram. Patients with coronary total occlusion were only divided into subacute (≤3 month), SD-CTO (3–6 month) and LD-CTO (≥6 month) groups in this study. However, the duration of the occlusion does not simply imply the time of complete organization of the thrombus in CTO. Furthermore, histopathology data were not available. Therefore, the correlation between the HIS and tissue characteristics remains unknown, and we should pay attention to the difference between occlusion duration and thrombus age. Third, as this study was of an observational nature, the utility of the HIS for a procedural success rate for CTO was not determined. The results of the PCI for CTO were influenced by selection criteria, experience of the operators, strategies and devices used. Therefore, further prospective randomized trials should be conducted to examine whether the presence of HIS in the occlusion site might be a predictor of successful PCI for CTO lesions. Fourth, in the present study, areas with TMR greater than 1.0 were defined as HIS. Although the optimal cut-off value of TMR was controversial, HIS with TMR greater than 1.0 showed close association with the presence of intracoronary thrombus in our previous report [9]. As the presence and aging of the intracoronary thrombus represented with HIS was one of the key factors in this analysis, the cut-off value of 1.0 was thought to be reasonable. Fifth, only MR angiography and T1WI were performed in the present study; however, additional sequences such as late gadolinium enhancement are ideal. Finally, the number of patients examined in the present study was very small.

## Conclusions

This study showed that the presence of a HIS in the occlusion site was significantly and more strongly associated with SD-CTO than LD-CTO among patients with CTO in whom the occlusion duration could be estimated. These results may help to estimate the occlusion duration in patients with CTO.

## Abbreviations

AMI: Acute myocardial infarction
CAG: Coronary angiography
CTO: Chronic total occlusion
HIS: High-intensity signal
LD-CTO: Long-duration chronic total occlusion
MR: Magnetic resonance
PCI: Percutaneous coronary intervention
SD-CTO: Short-duration chronic total occlusion
T1WI: T1-weighted imaging
TIMI: Thrombolysis in Myocardial Infarction
TMR: Signal intensity of the target area within a coronary artery to cardiac muscle ratio
